# Integration of Dementia Systems in Central America: A Social Network Approach

**DOI:** 10.5334/ijic.7630

**Published:** 2024-02-27

**Authors:** Nereide A. Curreri, Dave Griffiths, Louise Mccabe

**Affiliations:** 1 University of Applied Sciences & Arts of Southern Switzerland (SUPSI), CH; 2 University of Stirling, UK

**Keywords:** older adults, dementia, integration, services, LMIC

## Abstract

**Introduction:**

Action 3 of the UN Decade of Healthy Ageing plan is to deliver integrated care to improve older adults’ lives. Integrated care is vital in meeting the complex needs of people with dementia but little is known about how this is or could be delivered in low and middle income countries (LMIC). This paper provides insights into previously unknown care system structures and on the potential and reality of delivering integrated care in Central America for people with dementia.

**Methods:**

A social network analysis (SNA) methodology was adopted to engage with providers of services for older adults and families with dementia in Guatemala, El Salvador, Honduras, Costa Rica and Panama. Sixty-eight (68) semi-structured interviews were completed, 57 with organisations and 11 with families.

**Results:**

Across the five countries there was evidence of fragmentation and low integration within the dementia care systems. A variety of services and types of providers are present in all five countries, and high levels of diversified connections exist among organisations of differing disciplines. However, unawareness among network members about other members that they could potentially form active links with is a barrier on the path to integration.

**Conclusion:**

This innovative and robust study demonstrates SNA can be applied to evaluate LMIC care systems. Findings provide baselines of system structures and insights into where resources are needed to fortify integration strategies. Results suggest that Central American countries have the building blocks in place to develop integrated care systems to meet the needs of people with dementia, but the links across service providers are opportunistic rather than context based coordinated integration policies.

## Introduction

The United Nations (UN) Decade of Healthy Ageing plan (2020–2030) proposes four main action areas that guide stakeholders including governments to improve the lives of older adults. The third action area is to “Deliver person-centred, integrated care and primary health services responsive to older people” [1, p. 12]. One sub action for Member States to “Assess the capacity and readiness of the health system to implement Integrated care for older people (…)” [1, p. 13] resonates with Provan and Milward’s advice that before investing time and money into integrating services, an attempt should be made to establish how integrated a system is [2]. Thus, this study utilised a social network analysis to assess the levels of integration in five Central American countries, a region where little is known about systems of care for people with dementia. Dementia is an age-related condition that leads to a complex range of care and support needs for the person and their family, making the provision of integrated care particularly important.

### Background

Dementia is a complex, age-related condition that is primarily characterised by cognitive decline and that affects people across all aspects of their health and wellbeing [3]. Currently there is no medical cure and individuals and families rely on, when available, health and social support services to maintain wellbeing as the condition progresses [4]. A variable decline in functional abilities [5,6] creates biomedical, psychological, and social needs that can be best addressed through collaboration between health and social care sectors, that is, through integrated care [2,7,8,9].

Research on dementia services in high income countries finds that integration of health and social care, for example through dedicated one stop shop dementia integrated care centres, leads to increased access to multidisciplinary services and continuity of care for families [10,11,12]. The integration of services can also aid in reducing the treatment of dementia as an isolated condition, where comorbidities may influence care needs and processes [9]. For people with complex needs such as those with dementia, it is particularly difficult to navigate what are commonly fragmented care and support systems [13]. “A vivid and strong network of care providers is essential for delivering quality case management” for families with dementia [11]. From here on, the term families with dementia will be used to indicate the family and the person living with dementia as part of the family, rather than two separate entities.

The World Health organisation (WHO) and Alzheimer’s Disease International (ADI) as well as researchers estimate LMICs to be the future home to the majority of the world’s older adults and, consequently, to the majority of people with dementia [14,15,16,17,18]. Research conducted to date about dementia in LMICs, including data on formal and informal care arrangements, identifies a need to provide more robust data about dementia specific services [4,19]. Latin American countries persistently lack research in dementia. Central America is particularly under-represented and Central American states are often omitted in Latin American comparative studies [16,20]. The gaps in health services exposed and widened by the COVID-19 pandemic alongside continued increases in the prevalence of dementia in the Americas further underline the need to understand health system integration [21,22].

Bunn et al. highlight a lack of evidence about system structures and coordination of services for individuals with complex needs such as those with dementia [9]. Furthermore, de Carvalho et al. indicate that in LMIC and specifically Latin American models of care, system integration is rare, as is their evaluation [7,23]. In fact, the WHO, Blanchet and James, and Keating argue that understanding system structures, contexts, and processes of LMICs will help to design improved interventions [18,24,25].

This paper addresses the lack of evidence on dementia care within Central America, providing a cross-national comparative analysis of the integration of services in five countries. We present findings on the integration levels of the older adult service systems for families with dementia using an SNA approach. To the best of our knowledge, this is the first paper that portrays the structures of older adult service systems in any LMIC through a social network analysis [23].

## Methodology

### Integration

A care system is built on a network of relationships which support the provision of services [26]. Smooth service delivery, accessibility and quality are all associated with inter-agency partnerships therefore interorganisational cooperation may bring integration of activities, increased efficiency, and reduced costs [27]. In his seminal article, The Five Laws of Integration, Leutz explains how three levels of integration enable sectors and disciplines to work together [28]. Leutz describes these three levels of integration within care delivery systems: linkage, coordination, and full integration [8,28,29,30]. Linkage across a system allows for general basic screening knowledge and referral to the appropriate service provider, as well as the provision of information on care pathways and payments across providers. Coordination is achieved through structured processes that enable information sharing and managing transitions across sectors. The full integration level is achieved when providers share common goals and information systems and jointly develop new integrated service programs.

According to Leutz integration is: a) the connection of health care services to other human services as a means of improving efficiency and client satisfaction, and b) the formation of managed care organisations that provide health and social support or a closer coordination of individual care [28,30]. In the literature of integrated care and integration in dementia care, the divide between the health and social service sectors is indicated as one of the main causes of service fragmentation, resulting in low quality [2,7,9,31]. Leutz’s definition of integration includes the element of coordination of care, which the lack of is deemed to augment fragmentation of care. At the core of system integration is its impact on the effectiveness of service provision for those receiving support [32].

Although Leutz’s model does not provide a methodology to measure the three levels of integration, Blanchet and James suggest using social network analysis (SNA) when evaluating integration of health systems and specify which network properties to measure as indicators of integration [24]. They propose three steps to measure integration: the first is defining a list of actors in a network; the second is establishing the types of relationships between actors; and third is analysing the structure of the network by measuring network properties. The properties or integration indicators to measure are ‘density’ (the proportion of plausible connections which are observed with the network) and ‘centrality’ (the positioning of specific actors, or nodes, within the network). Through SNA, visualisations of the networks are created and the interconnections across actors are highlighted, and together with the indicators, levels of network or system integration can be assessed.

Such approaches are commonplace in the analysis of high-income countries and have rarely been applied in LMIC countries [24,33,34]. Following Blanchet and James’ framework, social network analysis was used in this study to illustrate the structure of existing relationships among organisations providing older adult support services to families living with dementia in each country, and to measure and compare integration across the countries.

### Study Setting/Context

Central America is home to approximately 180 million people. Guatemala is the most populated country and Belize the smallest (Table 1). Adults over 65 years old make up between 5–10% of Central American countries’ populations, with a steady increase [35]. Guatemala, El Salvador, Honduras, Costa Rica, and Panama were included in this study. Belize was not included because ethical approval from each potential service provider before fieldwork was advised, which the sampling method could not support. Nicaragua was excluded due to civil/political unrest that developed into a revolution with the closing of its borders at the time of fieldwork.

**Table 1:** Population in Central America.


	CENTRAL AMERICA	GUATEMALA	EL SALVADOR	HONDURAS	COSTA RICA	PANAMA	BELIZE	NICARAGUA

Population* (thousands)	177,587	17,581	6,454	9,746	5,048	4,246	390	6,546

**Population** > 65 y.o.*	7%	5%	8%	5%	10%	5%	5%	8%


*UN population prospects data 2019; highlighted countries included in study.

The national health systems are generally polarised into the public and the private sectors. Health coverage is ranked high for basic health services in Central America [36]. Laws specific to protecting older adults’ rights were developed beginning in the 1990s. These special laws are extremely valuable as they allow the state to organise action in favour of older adults and establish limits and possibilities for public authorities to exercise older adults’ rights [37]. As of 2016, every Central American country has a law regarding older adults included in their constitutions. Yet, most countries in the region lack specific policies for people living with dementia [38,39]. Costa Rica is the only country of the region that has a national dementia plan published in 2014, and El Salvador has a plan awaiting ratification.

Alzheimer’s Disease International has a regional branch, Alzheimer Iberoamérica, that acts as the federation of Alzheimer’s associations and foundations of the 21 Ibero-American countries [40]. A national Alzheimer’s association is present in each country of the Central American isthmus, not all of which were operational at the time of the fieldwork, as discussed in the findings that follow.

### Methods

#### Ethics and recruitment

Ethical approval was obtained from our university’s ethical committee. Verbal informed consent was selected for this project because of the cultural and socio-political context which could have influenced an individual’s willingness to sign papers and/or be audio recorded as noted in the Canadian Sociological Association’s Statement of Professional Ethics point number 21, supported by Brijnath’s research in a developing country [41,42]. Creed-Kanashiro and Hyder and Wali also recommend flexibility in documenting written informed consent when conducting research in developing countries allowing for alternatives such as verbal and/or community informed consent practices [43,44].

Participants were recruited using reputational and snowball methods. Beginning with the Alzheimer’s associations in each country, the representatives of each organisation were asked to name a set of organisations they either collaborated with or deemed as service providers for families with dementia. In the successive interviews, snowball sampling was employed to enlist additional organisations which provided services for families with dementia and to confirm network affiliation. Snowball sampling was considered the best approach as it enabled sampling to follow the network as it was experienced by organisations in each country. Criteria for inclusion in the study were providing any type of service to people living with dementia and/or their families. Families with dementia were also recruited through the Alzheimer’s association in each country. This follows Waite’s description of families as founding social institutions enabling an active service recipient and provider perspective to be incorporated within the snowballing of organisations providing services [45]. Acknowledging families as active participants in care provision of their loved ones rather than passive users also allows their gatekeeping role to emerge. Methodologically, families nominated organisations which weren’t central therefore causing little substantive changes to the data. Their inclusion doesn’t provide a different interpretation, but does enable this research to be inclusive and reflect the lived experience of some families in each country. Many of the organisations identified in the sampling process provided support for older adults while specialist services for people with dementia were rare.

#### Data collection, and analysis

Data collection was completed through face-to-face interviews (60 minutes on average) with a key informant provided by each institution. Sixty-eight (68) semi-structured interviews were completed (Table 2), eleven of which were with families with dementia, and the remaining with representatives of organisations providing services to older adults and/or families with dementia. Only one interview was conducted with each organisation. It was not always possible to interview representatives of each nominated organisation, but they have been included within the analysis as they form part of the wider network.

**Table 2:** Number of interviews and organisations nominated.


	GUATEMALA	EL SALVADOR	HONDURAS	COSTA RICA	PANAMA	TOTAL: 5 COUNTRIES

**n. interviews organisations**	14	14	12	8	9	57

**n. interviews families**	3	2	2	2	2	11

**n. nominated organisations**	37	26	17	23	10	113


Interviews were semi structured and began with a request for a brief background on the organisation the participant represented, followed by a request for a list of organisations they have been or are in contact with. All types of contact between organizations are encompassed in this step of creating the networks. The items were developed based on integration indicators, SNA survey and questionnaire examples found in the literature and the SNA Resources website [46,47]. Subsequently, qualitative data about the organisations and quantitative data about network connections were grouped separately for distinct analyses.

##### Indicators and measurements

Interview data was converted to matrices illustrating which organisations were connected to which others. Specialised software, UCINET (Borgatti et al. 2002) and Pajek (Batagelj and Mrvar 2014) were used to calculate sociograms and summary statistics about the network for each country [48,49]. As noted, interconnecting the various sectors of a health system improves outcomes for users [28,50]. Thus, social network analysis was applied to the data to explore those interconnections and assess the levels of integration across three network properties: density, closeness centralisation, and E-I index.

Density measures the proportion of possible connections observed within a health care system. Access to a variety of organisations through interconnections between disciplines and sectors within a health care system fosters multidisciplinary and efficient communication, key elements of integrated care and system integration. The higher the density, or the more connected organisations are to one another, the faster exchanges such as information can flow through the network [47]. Valente et al. identified a “Goldilocks Principle” where just the right amount of density must be determined [51]. Between 30% (.30) and 50% (.50) of possible ties being formed provides the right balance between being too disconnected to cooperate and being too well connected to disseminate effectively [52].

Closeness centralisation measures the fewest number of intermediaries passed which allows all network members to pass information to all others [53]. This presents an indicator of the level of managed coordination, a key element of integration and integrated care and a sign of greater coordination of the network [54,55]. Closeness centrality shows the relative position of each actor by measuring how few intermediaries are needed for one organisation to pass information to all others. The organisation with the shortest path, or closest to every other organisation in the network, will have the highest closeness centrality score. Node (organisation) closeness centrality scores range from 0 to 1, where 1 indicates that a node is connected directly to all other nodes.

Finally, the E-I index measure addresses the wide range of care interventions required in an integrated approach to care [8,56]. Organisations have been broken down into groups based on their industry type, such as hospitals, nonprofits, and private businesses. This scheme, shown in Table 3, was based on self-reported descriptions by organisations in the interviews. Whilst there are some crossovers (nonprofits and universities, for instance), the schematic presents the predominant sector as described by respondents across the region. The number of ties an organisation has external to its group is subtracted by the number of ties that organisation has internal to its group and then divided by the total number of ties it has [57]. A rescaled parameter (the E-I index) ranges between having all ties external to your type (+1) and all ties internal within your type (–1) [57]. More external ties imply environments with a range of connections, multi-disciplinary and multi-sectoral, allowing organisations to meet complex needs as put forward by the integration literature [2,9,58].

**Table 3:** Total number of organisations by type, country, colour code.


ORGANIZATION	CC	GUATEMALA	EL SALVADOR	HONDURAS	COSTA RICA	PANAMA	TOTAL

Alzheimer Association		1	1	1	1	1	5

Care Homes		11	4	3	1	2	21

Families		3	2	2	2	2	11

Government		12	17	8	11	4	52

Hospital		9	4	4	3	0	20

Non-profit		6	4	3	5	2	20

Private		5	6	4	5	4	24

Social Security		4	1	1	3	1	10

University		3	4	3	3	3	16

Total		54	43	29	34	19	179


cc = color code.

## Results

The results present comparisons between the countries on key measures of density, closeness centrality and the E-I index, followed by a more detailed presentation of each country.

### Whole network characteristics

Overall, government and private organisations emerged as the most frequently nominated service providers (Table 3). Government organisations included ministries, municipalities, public dormitories, public day centres, military hospitals and or day centres and pensioners’ associations. While private enterprises are types of organisations that are for profit and provide services to older adults for a fee.

### Network properties

Table 4 displays the scores for density, closeness centralisation and E-I index by country and rank. Following on the seminal work on interorganisational networks by Provan and Milward, the networks within each indicator have been ranked (1–5) in order of highest to lowest score [2]. The cross-country comparisons offer context for the scores amongst other networks.

**Table 4:** Network properties by country.


COUNTRY	GUATEMALA		EL SALVADOR		HONDURAS		COSTA RICA		PANAMA	
					
*TOTAL NETWORK*	*N = 53*		*N = 41*		*N = 29*		*N = 31*		*N = 27*	
					
*NETWORK PROPERTY*	SCORE	RANK/TYPE	SCORE	RANK/TYPE	SCORE	RANK/TYPE	SCORE	RANK/TYPE	SCORE	RANK/TYPE

Density	.039	5	.056	2	.045	3	.043	4	.083	1

Centralisation	.524	1	.386	3	.305	4	.456	2	.267	5

Highest Centrality	.658	A	.55	A	.489	AH	.556	A	.5	AH

E-I index	.535	4	.357	5	.676	1	.6	3	.647	2


A = Alzheimer association, AH = Care Home.

Generally, the results demonstrate very low linkage levels in all countries; barely 10% of possible ties were actualized. This points to generally very low integration levels. Reiterating the Goldilocks principle, an ideal density measure is between .30 and .50 where an adequate amount of sharing is allowed for, while shielding from an overwhelming quantity of information transfer that may be burdensome and clog coordination [51]. Low and high density both hinder cooperation by creating barriers to the flow of information and resources due to lack of capacity, too few or too many ties preventing effective integration. Density scores emerged between .039 and .083, depicting low proportions of connections across the organisations, preventing knowledge and information sharing, referrals, and multidisciplinary collaboration.

The most centralised networks emerged in Guatemala and Costa Rica where it takes an average of around two steps (.4 to .5) to get to the other organisations. In both countries the Alzheimer’s associations had the highest closeness centrality, linking to a wealth of government agencies. This produced networks which were better connected, offering more opportunities for levels of coordination where information and knowledge of services is harmoniously controlled and interorganisational cooperation is guided. This demonstrates the importance of a strong infrastructure organisation at the heart of the sector, with connectivity to policymakers, creating the opportunities for information and resources to flow through to service providers.

By contrast, low centrality emerges in El Salvador, Honduras, and Panama, (.267–.386) indicating coordination amongst a few organisations but the lack of involvement of the network at large. The coordination of the Alzheimer’s associations was not as apparent in these countries. In Panama and Honduras, it was care homes which had the highest closeness measure. In El Salvador the Alzheimer’s association was the most central but generally lacked connectivity to government agencies reducing opportunities for collaboration and closer integration.

E-I index results were between .54 and .68 indicating moderate levels of external ties for all countries. A higher score indicates greater diversity of relations between groups, implying multi-disciplinarity and multi-sectoriality, which indicates a higher level of integration. This suggests that a willingness exists for organisations to make connections across the sector. The exception to these scores was in El Salvador, with a more moderate score of .36, due to stronger links between government agencies than seen elsewhere in the region but without that leading to increased connectivity between, or to, other types of organisations.

### Network indicators by country

A sociogram for each country provides a visualisation of the national inter-organisational connections across the older adult service networks that provide services for families with dementia.

Interorganisational ties are presented for each country. The circles represent an organisation or node, every colour a different type of organisation, and the arrow a relationship between two actors. The arrow shoots from the organisation reporting the tie and points to the organisation they nominated.

The sociograms are explained below individually relative to the network property measures that indicate proportion of connections, coordination, and multisectorality. The network properties density, closeness centrality, and E-I index are described in detail in the following section where the country networks are presented in order of highest to lowest density score.

**Figure 1:**
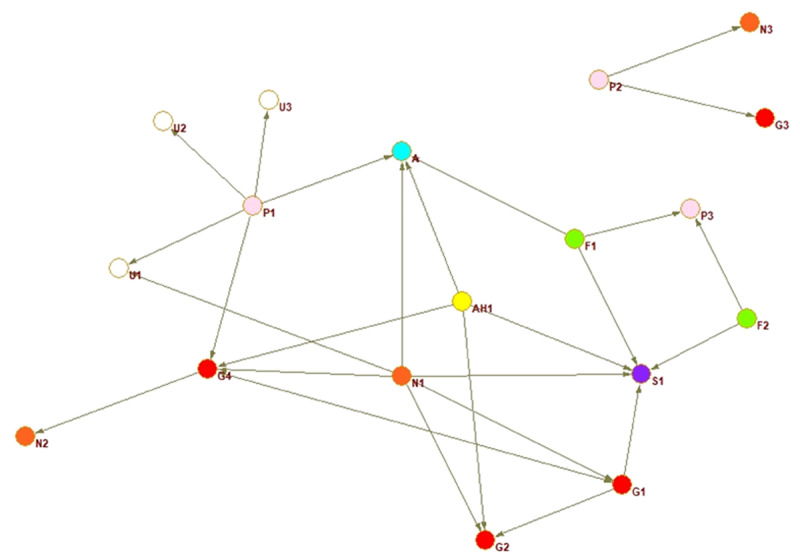
Panama whole network.

Panama’s network (Figure 1) includes one separate component disconnected from the rest of the network. Separate networks or components, emerge when organisations are nominated because they are known to exist, but in practical terms, they do not have relations, they are not connected to the main network. A care home is the most central node having the most ties to other organisations yielding coordination power. Density is low, a barrier to multi-disciplinary and multi-sectoral collaboration. Neither of the two networks are at an appropriate level in the three network properties to sustain linkage and coordination, two primary factors in achieving integration. Density is low, and centralisation is operationalised differently throughout the region, but intersectorality is present depicting fragmentation at the whole network level rather than at the sector level.

**Figure 2:**
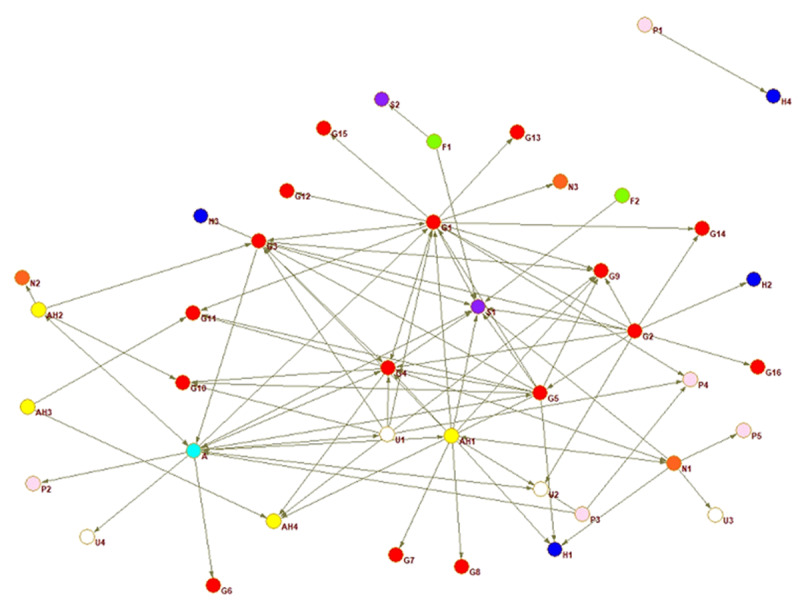
El Salvador whole network.

In El Salvador (Figure 2), the Alzheimer’s association plays a central role connecting multiple nodes, Guatemala’s network, presented below, shows something similar; nodes surround the core of the network with one tie connecting them to the rest of the organisations. The closeness centrality of the Alzheimer’s association was lower than in Guatemala, with many interviewees external to the association stating they offered a dearth of services and were not well known. The centralisation score was relatively lower than other countries, meaning it was more complicated to get information transmitted across the network.

**Figure 3:**
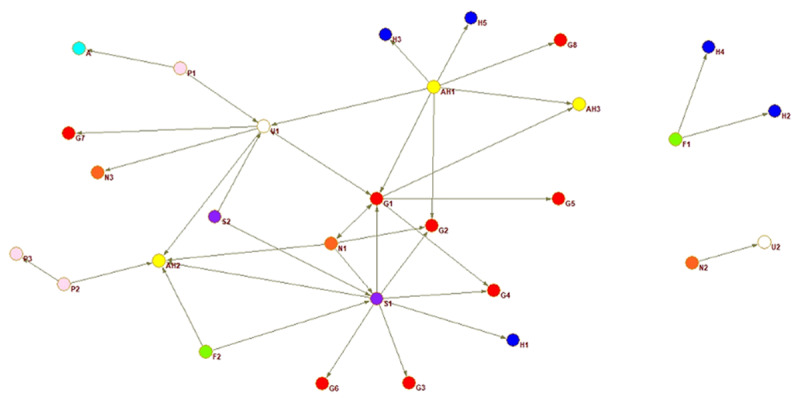
Honduras whole network.

Honduras’ network is visually fragmented (Figure 3), with two separate components not connected to the main network. Three groups appear better connected, with a university, a care home, and a social security institute as central nodes. A government organisation connects the three parts of the main component, which are not directly interconnected. Exchange might flow easily through the main network, where the groups are connected with each other, yet in general density is low confirming fragmentation. The Alzheimer’s association is in a more peripheral position and there is no real centre to the Honduras network.

**Figure 4:**
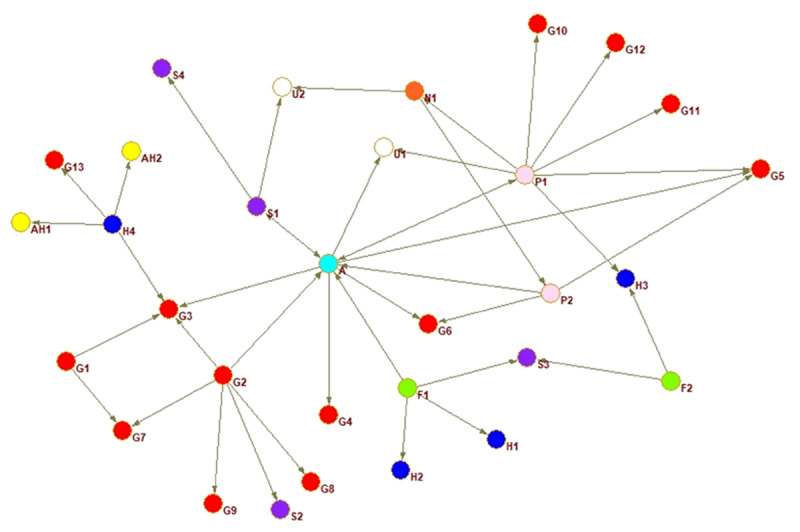
Costa Rica whole network.

In Costa Rica, the Alzheimer association is the most central node, bringing together the network and linking together many otherwise unconnected strands of organisations. Whilst the closeness centralisation score is relatively high, this is due to the Alzheimer association connecting many fragmented groups, with network density being relatively lower than other countries. Figure 4 shows (red) government agencies generally coagulating in two separate sides of the sociogram and with few ties to each other.

**Figure 5:**
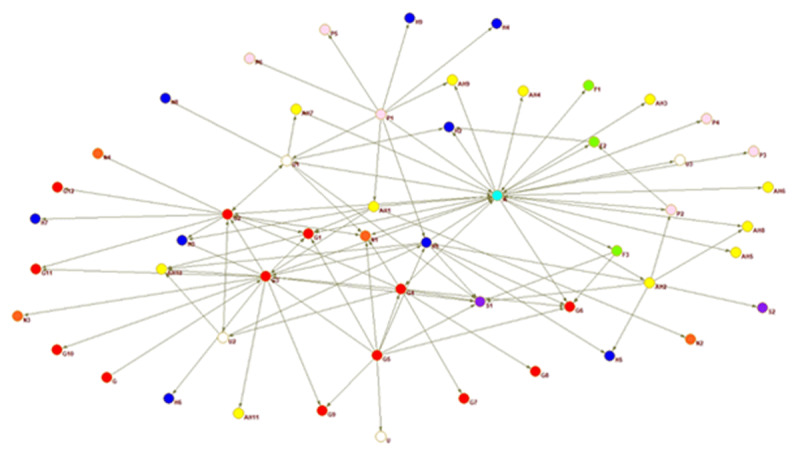
Guatemala whole network.

In Guatemala (Figure 5) the (light blue) Alzheimer’s association is in the central position, highly centralised indicating a coordination role. Many nodes are on the outskirts of the network and have only one tie connecting them to the network. The visual portrays a grouping of organisations; where on one side a concentration of government organisations and on the other side care homes, families and private organisations are gathered. Sorting such as this occurs as similar organisations are either linked to each other or linked to the same organisations. The sociogram portrays a trend of government organisations to be more in contact with each other than with other types of organisations, while the private companies and care homes are tied to the same organisations. The density measure (see Table 4) shows that only 4% of possible ties are realised, indicating a fragmented network where organisations serving the same individuals do not interact.

## Discussion

This study shows the successful application of social network analysis (SNA) to assess the integration levels of five Central American older adult service networks currently supporting families with dementia. The primary data derived through SNA provides a unique outlook on how system level evaluation can inform providers and policy makers on care provision for people with complex needs such as people living with dementia.

These Central American networks may boast a variety of types of organisations and services but if interconnectedness is absent, the capacity of the networks to supply continuity of care is limited [2,28]. Nevertheless, the E-I index scores showing the available access to a variety of organisations denote the existence across the five country networks of interconnections between disciplines and sectors, fostering multidisciplinarity, a key element of integrated care and system integration. The availability of accessing a variety of actors responds to calls for intersectoral action and enables finding “multi-scale solutions to multi-scale problems” such as complex needs from dementia and comorbidities [24,59]. These findings provide a context of baseline diversity within the networks, indicating underpinnings for integration.

Service providers were identified in each country that were previously unknown among network participants, as were their connections or lack thereof. Interviewees were observed to be unaware of most organisations providing services to the same cohort, often reacting with surprise and desire to be connected. The results of the snowball sampling are reflected in the sociograms, where few organisations were cognizant of or collaborating with other providers. Low density scores confirm obliviousness of an existing network and participants, and underline the presence of needs-based connections where formal partnerships or collaboration agreements are missing [60]. This suggests significant untapped potential in these networks for more integration and interdisciplinarity, both called for by Robledo et al. who describe that the need for new approaches to integrated care delivery has been augmented by Covid [23]. Dementia is a leading cause for health and care services needs globally, which makes addressing insufficiency and fragmentation of services essential [61].

Linkage levels are low but demonstrate multisectorality, and coordination is moderate in two of the five networks. Returning to Leutz’s three levels of integration, El Salvador, Honduras, and Panama emerge with low levels of both linkage and coordination underlining networks where organisations work independently. While Guatemala and Costa Rica demonstrate efforts in coordination of the low proportion of connections. The presence of coordination even across few connections facilitates sharing of information and thus decreases fragmentation in care provision [28,30]. Higher linkage would allow for screening and needs identification, as well as referrals to appropriate multisector services, both strategic elements of person-centred care [62]. More evident coordination levels across the networks would indicate accountability through assigning responsibility for case management across disciplines, with the aim of addressing families’ needs in a timely manner, such as implementing home care at discharge from hospital, rather than the family struggling to find support. The low coordination levels of the Central American service systems prove a lack of organised relationships. If organised arrangements sustain integrated care, as Pieper claims, then the service networks of this study will be unable to provide integrated care [63]. Arrangements such as interorganisational agreements or contracts appear to be missing given the low linkage and coordination in the Central American systems investigated here.

Findings represent a conducive social environment at a linkage level of integration, where organizations can connect with each other, with a need for structure to allow information to flow across the network. Based on Freeman’s concept of centrality and de Nooy and Batagelj’s description, the organisations within the networks have few ties, meaning they interact with few others, and can influence and be influenced by only a few in the network [53,64]. This points to a predominance of independent ties undermining multidisciplinary collaboration and a need for a central reference point to take leadership of the network and coordinate service provision.

## Limitations

A few limitations of this study should be considered when interpreting its findings. Non-response of some organisations might have shaped the networks differently if they had nominated ties. One representative of each organisation was interviewed, precluding any ties between other employees and other organisations. In addition, ties were established via one organisation’s claim, where possible the claims were verified, but not all organisations nominated were interviewed due to time and resource constraints.

## Implications for research and practice

The findings of this study may influence policies on and implementation of partnership working and new inter-organizational practices leading to outcomes of increased system integration and consequently increased provision/receipt of integrated services. Older persons and families with dementia in LMICs must often confront either scarcity in services or high-cost services, differently from many high-income countries where universal health care is common [65].

In the Central American countries studied, the ubiquity of governmental organisations shows a dominance in service provision. Their strong presence makes them valuable collaborators, particularly for families with dementia through their consistent ties to the Alzheimer’s associations, as well as potential coordinators in the networks. Linkage and coordination strategies could increase network cooperation through cross-referrals decreasing the pressure and costs for the public governmental agencies.

Care homes and Alzheimer’s associations are the most central nodes across the networks, thus considerations for resource allocation to these types of services could sustain coordination. Efforts to take on roles of responsibility such as network leadership and coordination infer trained and dedicated professionals, time, and infrastructure. Funding opportunities for these already established central actors could sustain the coordination level for network integration.

Central American service providers can use these findings as an invitation to build their capacity within their national networks. By connecting to the other service organisations, the network is enriched through arranged collaboration across disciplines. Shared development of network coordination leads to system integration and families’ complex needs are thus met through integrated care by way of the network of connections.

At the whole network level, structural indicators can be compared cross-nationally making the transferability of this study to any context raise the same research questions for other countries. Key policy integration indicators for evaluating possible gaps in care service provision systems and to monitor the development of integration can be found using the methodological framework of this study. Finally, the design of context-based interventions supporting cooperation is streamlined through this type of evaluation [66].

## Conclusion

This research pioneers with primary data of older adult and dementia services in five Central American countries missing from the current literature. The findings indicate that the five countries have similar, multidisciplinary and multisectoral systems. An overall low level of integration was assessed for each country due to very low density and inconsistent coordination.

To enable countries to deliver integrated care as advocated in the UN Decade plan action 3, an initial assessment of the existing system using SNA can provide a picture of its structure, a map of services/providers, their interconnections, and any gaps. Furthermore, in LMIC where there is limited data on health systems, the exploration of interorganizational relations aids in gathering primary data on what services are being offered, who is offering them, where there is collaboration, and the gaps; offering a route to integrate care that improves the lives of people with dementia and their families.
